# A Review of the Impact of Alterations in Gut Microbiome on the Immunopathogenesis of Ocular Diseases

**DOI:** 10.3390/jcm10204694

**Published:** 2021-10-13

**Authors:** Yashan Bu, Yau-Kei Chan, Ho-Lam Wong, Stephanie Hiu-Ling Poon, Amy Cheuk-Yin Lo, Kendrick Co Shih, Louis Tong

**Affiliations:** 1 Department of Ophthalmology, Li Ka Shing Faculty of Medicine, The University of Hong Kong, Hong Kong, China; u3005204@connect.hku.hk (Y.B.); josephyk@connect.hku.hk (Y.-K.C.); whlww@connect.hku.hk (H.-L.W.); stephaniehlpoon@gmail.com (S.H.-L.P.); amylo@hku.hk (A.C.-Y.L.); 2 Cornea and External Eye Disease Service, Singapore National Eye Centre, Singapore 168751, Singapore; louis.tong.h.t@singhealth.com.sg; 3 Ocular Surface Research Group, Singapore Eye Research Institute, Singapore 169856, Singapore

**Keywords:** ocular disease, intestinal microbiota, autoimmune diseases, immune homeostasis

## Abstract

Recent studies have highlighted the association between ocular diseases and microbiota profiles of the host intestinal tract and oral cavity. There is mounting evidence supporting the existence of a ‘gut–eye axis’, whereby changes in gut microbiome alter host immunity, with consequential implications for ocular health and disease. In this review, we examined recent published findings on the association between gut microbiome and ocular morbidity, based on 25 original articles published between 2011 to 2020. The review included both clinical and in vivo animal studies, with particular focus on the influence of the microbiome on host immunity and metabolism. Significant associations between altered intestinal microbiome and specific ocular diseases and pathological processes, including Behçet’s syndrome, autoimmune uveitis, age-related macular degeneration, choroidal neovascularization, bacterial keratitis, and Sjögren-like lacrimal keratoconjunctivitis have been demonstrated. Furthermore, alterations in the gut microbiome resulted in quantifiable changes in the host immune response, suggesting immunopathogenesis as the basis for the link between intestinal dysbiosis and ocular disease. We also examined and compared different techniques used in the identification and quantification of gut microorganisms. With our enhanced understanding of the potential role of gut commensals in ophthalmic disease, the stage is set for further studies on the underlying mechanisms linking the gut microbiome, the host immune response, and the pathogenesis of ophthalmic disease.

## 1. Introduction

The microbiome refers to a community of microorganisms and the sum of their genetic material. In the last decade, the concept of the ‘gut–eye axis’ has been brought to greater attention, with a recent surge in studies investigating the role of the intestinal microbiome in specific ocular diseases, including autoimmune uveitis, age-related macular degeneration (AMD), and Behçet’s syndrome (BS). There is now robust evidence supporting the impact of intestinal dysbiosis on ocular morbidity. Therapeutic strategies targeting the intestinal microbiome are promising alternative methods to conventional treatments for ocular inflammatory diseases [1]. Such treatments include the use of antibiotics, introduction of specific microbes, use of probiotics, and fecal transplant therapy [2].

The interaction between microbial components or products and the host innate and adaptive immunity in the pathogenesis of ophthalmic disease has been reported in the published literature [3]. Specifically, a clear association between ophthalmic inflammatory disease and the intestinal microbiome is illustrated by the relationship between acute anterior uveitis and specific spondylarthritis subtypes; ankylosing spondylitis, reactive arthritis, and psoriatic arthritis [4]. Recent studies have examined the interactions between microbiota and the immune system on a molecular level and across a number of ophthalmic diseases. Here, our review summarizes recent studies on the topic, published within the past 10 years. With this information, we aim to explore future directions for clarifying gut-influenced immune responses that in turn play a role in the pathogenesis of ophthalmic diseases, as well as the potential for manipulation of intestinal microbiome as a therapeutic strategy for specific ocular disorders.

**Figure 1:**
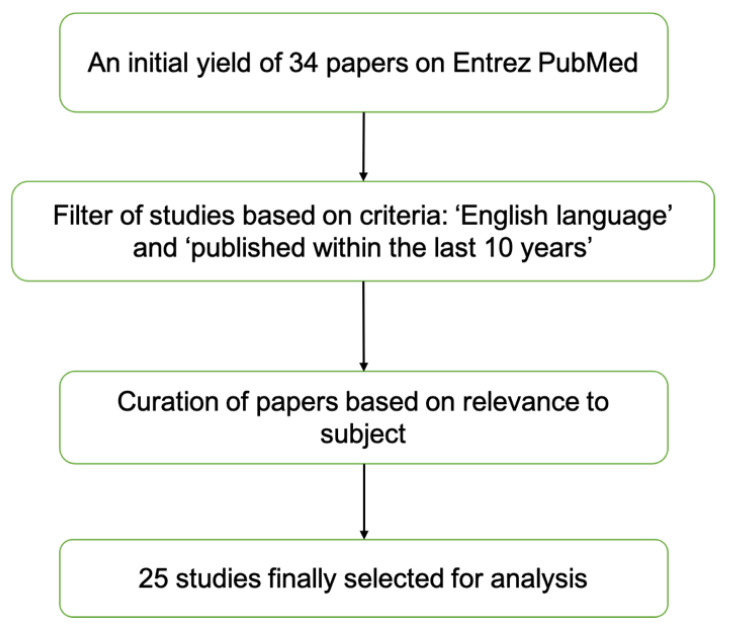
Flow chart for paper search strategy.

An Entrez PubMed search was conducted on 17 February 2021 using the search terms “microbiome”, “ocular”, “eye”, “ophthalmic”, “immune”, and “immunity”. Only articles written in English and published within the past 10 years were included. Thirty-four articles were initially identified using the above criteria. The resulting articles were then manually curated for relevance by Y.B. and K.C.S. For example, papers concerning the intestinal/ocular microbiome, systemic/ocular immunity, and ocular disorders were considered as relevant. Furthermore, only original articles were included, excluding review papers and meta-analyses. This produced a list of 18 relevant articles (see Figure 1). Additionally, 7 studies were identified in the references of the finalized results to give a total of 25 papers for the review. The fundamental information and significant findings of the studies are presented in Table 1.

## 2. The Association between Microbiome Profile and Ocular Disease

### 2.1. Behçet’s Disease

Behçet’s disease is a systemic vasculitis characterized by muco-cutaneous and ocular manifestations and involves the central nervous system and vascular and gastro-intestinal systems [14]. By comparing the fecal microbiota of 22 Behçet’s disease patients with that of 16 healthy co-habiting controls, it was found that the genera *Roseburia* and *Subdoligranulum* were in lower abundance in the fecal microbiota of Behçet’s disease patients [5]. Researchers found, for the first time, consistent and specific changes in the microbiome profile in patients with Behçet’s disease. Furthermore, a significant reduction of gut microbial butyrate production was detected in Behçet’s disease patients, which in turn may account for the reduction in regulatory T cell (Treg) responses and activation of T-effector responses. Butyrate is a fatty acid essential in the promotion of Treg cell differentiation [15]. From published studies, interferon-γ (IFN-γ) and interleukin-17 (IL-17) producing T-lymphocytes are thought to act as the main effector cells, together with neutrophils, in the pathogenesis of Behçet’s disease [5]. Therefore, gut dysbiosis and decreased butyrate production in Behçet’s disease may play a role in disease pathogenesis by directly causing dysregulation of T-cell responses.

### 2.2. Autoimmune Uveitis

Uveitis is an inflammatory condition of the eye affecting various ocular tissues, including the iris, ciliary body, choroid, retina, optic nerve, and vitreous humor [16]. It is associated with systemic autoimmune syndromes such as sarcoidosis and Vogt–Koyanagi–Harada disease. In a recent experiment, an R161H mouse model of uveitis was used to study natural precipitators/triggers of the disease [6]. It was discovered that a microbiota-dependent signal activates retina-specific T-cells in the gut lamina propria of experimental mice prior to the onset of autoimmune uveitis. These T cells have the potential to secrete IL-17A, which was previously identified as part of the pathogenic process in autoimmune uveitis [17,18,19]. In another study, gut commensal microbiota facilitated the development of T helper-17 (Th-17) cells [20]. Both studies compared mice under specific-pathogen-free and germ-free conditions, without recording the specific changes in the microbiota. In the experiments, elimination of commensals, through the germ-free environment, led to a reduction in Th-17 cell activation in the intestine and a resultant attenuation of uveitis severity. Compared with polyclonal T-cells, which are not retina-specific, gut R161H T-cells preferentially exhibited a Th-17 phenotype, thereby playing a key role in autoimmune uveitis regulation [6].

Another study investigated the role of oral antibiotics treatment in the management of autoimmune uveitis [7]. Experimental autoimmune uveitis (EAU) was induced in mice with the use of interphotoreceptor binding protein peptides. The combination of oral ampicillin, neomycin, metronidazole, and vancomycin was effective in reducing disease severity in EAU, whereas no significant decrease of EAU severity was observed after intraperitoneal injection of antibiotics. Oral antibiotics treatment significantly reduced gut Firmicutes and Bacteroidetes abundance, as well as that of the bacterial class, Alphaproteobacteria. The study further noted that lymphoid tissue and retina Treg cell count increased in oral antibiotic-treated EAU mice compared to controls. Furthermore, IL-17, IL-2 production, and effector T lymphocytes were reduced in oral antibiotic-treated mice but not in controls. Thus, by remodeling intestinal microbiome and increasing Tregs in the gut and extraintestinal tissues, oral antibiotic treatment may have a role in modulating disease severity in EAU. This finding suggests the presence of potentially uveitogenic intestinal microbiota, that may serve as identifiable risk factors for autoimmune uveitis. However, it is important to note that the oral antibiotic combination in experiments may have also induced changes in the gut mucosa as well as unrecorded alterations in the microbiota.

### 2.3. Age-Related Macular Degeneration (AMD)

AMD is a degenerative retinal disease characterized by atrophy of the retina pigment epithelium and photoreceptors, causing progressive central vison loss, particularly in the elderly [16]. It is the leading cause of blindness in the aged population over 60 in the Western world. In one study, an association was found between AMD and intestinal microbiome at the taxonomical level. Sequencing of gut metagenomes revealed distinct gut microbiome profiles between AMD patients and controls. Significantly higher abundance of *Anaerotruncus, Oscillibacter, Ruminococcus torques,* and *Eubacterium ventriosum* were found in AMD patients, whereas *Bacteroides eggerthii* was detected in higher abundance in healthy controls [8]. By examining the metabolic functions and pathways related to the altered intestinal microbiome, a reduction in bacteria responsible for fatty acid elongation, and an increase in bacteria responsible for L-alanine fermentation, glutamate degradation, and arginine biosynthesis were found in AMD patients. Glutamate, arginine, and long chain polyunsaturated fatty acids may play roles in retina physiology as well as in the pathogenesis of AMD. In addition to other studies demonstrating the link between AMD and nutritional factors [21,22,23], this finding suggested potential metabolic pathways by which the intestinal microbiome may impact disease progression in AMD [8].

A study performed shotgun metagenomic analysis of the intestinal microbiome of 57 neovascular AMD subjects and 58 healthy controls. The study also analyzed the intestinal microbiome of 16 complement C3-deficient mice and 16 wildtype controls using the same sequencing technique [9]. It was found that the class *Negativicutes* was significantly more abundant in AMD patients, whereas the genus *Oscillibacter* and the phylum *Bacteroides* were significantly more abundant in healthy controls. Moreover, the phylum *Firmicutes* was found to be more abundant in patients with neovascular AMD. The study identified a positive correlation between *Negativicutes* abundance and complement factor H and a negative correlation between *Bacteroides* abundance and complement factor H. The complement system is a key part of the innate immune system and is heavily involved in the pathogenesis of inflammatory and degenerative disease, including AMD [24]. Certain complement factor H polymorphisms have been strongly linked to increased vulnerability to the development of AMD and neovascular complications. This study uncovered a potential interconnection between the intestinal microbiome and the complement system in neovascular AMD. Yet whether AMD leads to the alterations in intestinal microbiome or whether the pathogenesis of AMD involves changes in the host gut microbiome, which in turn results in uncontrolled activation of the complement system, still remains unclear.

Choroidal neovascularization (CNV), a pathognomonic feature of exudative AMD, is represented by newly formed blood vessels originating from the choroidal circulation and proliferating either between the Bruch’s membrane and the retinal pigment epithelium (RPE) space (type 1) or between the retina and RPE (type 2) via breaks in the Bruch’s membrane [16]. High-fat diets (HFD) were found to exacerbate CNV by altering gut microbiota, particularly the proportions of Bacteroidetes, Firmicutes, and Proteobacteria, which may partly explain the known phenomenon of obesity-driven CNV [10]. HFD was also shown to augment the recruitment of microglia and macrophages during the development of CNV. Meanwhile, HFD results in intestinal dysbiosis, which in turn leads to increased intestinal permeability, elevated circulating levels of IL-6, IL-1β, tumor necrosis factor (TNF)-α, and vascular endothelial growth factor (VEGF)-A, and the expression of inflammation-associated mRNAs, which ultimately exacerbates pathological angiogenesis. Additionally, microbiota transplantation from regular diet-fed mice to HFD-fed mice was able to reverse intestinal dysbiosis and ameliorate the more severe CNV phenotype [10]. Clinically, this study highlights the role of intestinal microbiota in the progression of AMD.

### 2.4. Sjögren Syndrome (SS)-like Lacrimal Keratoconjunctivitis

SS-like lacrimal keratoconjunctivitis manifests clinically as dry eye disease, with corneal barrier disruption, conjunctival goblet cell loss, lymphocytic infiltration, and lacrimal gland epithelial apoptosis [12]. Commensal bacteria have a crucial role in immune homeostasis on the ocular surface and lacrimal glands [12]. In one study, the phenotypes of the eyes and lacrimal glands of germ-free and conventional C57BL/6J mice were compared, and it was found that germ-free mice had spontaneous lacrimal keratoconjunctivitis, comparable to the disease phenotype observed in patients with Sjögren syndrome. This result demonstrated that a germ-free environment, and thereby a loss of commensals, increased the frequency and production of IL-12 by antigen-presenting cells in the lacrimal gland functional unit. A fecal microbiota transplant was able to reverse lacrimal keratoconjunctivitis in germ-free mice, indicating that the disrupted corneal barrier function and lower goblet cell density observed at the ocular surface are directly related to the loss of commensal bacteria. Another study demonstrated that intestinal dysbiosis, driven by low relative abundance of commensal bacteria and high relative abundance of potentially pathogenic genera, was related to worse ocular mucosal disease both in both mice with SS-like keratoconjunctivitis as well as human SS patients [13]. The combined findings highlighted the importance of gut commensals in maintaining the ocular immune homeostasis, and the potential role in their depletion in developing autoimmune keratoconjunctivitis.

### 2.5. Bacterial Keratitis

Bacterial keratitis is a severe inflammatory disease of the cornea caused by bacterial infection, which is a significant cause of corneal blindness worldwide. In one study, it was found that alterations in gut bacterial and fungal microbiota were associated with increased susceptibility to bacterial keratitis [11]. All healthy controls and bacterial keratitis subjects in the study were recruited from Telangana, a state in South India. Fecal samples were collected and the 16S rRNA sequencing technique was used for microbiome analysis. The authors of the study reported a reduction in Firmicutes abundance in the gut of bacterial keratitis patients compared to that of healthy controls. Additionally, eight families of bacteria affiliated to the phylum Firmicutes, namely *Veillonellaceae, Ruminococcaceae, Lachnospiraceae, Clostridiaceae, Lactobacillaceae, Turicibacteraceae, Peptococcaceae, and Gemellaceae*, were significantly more abundant in heathy controls than in bacterial keratitis patients. Furthermore, significant variations were also observed in four low abundant phyla *Cyanobacteria*, *Elusimicrobia*, *Tenericutes,* and *Saccharibacteria*. Higher abundance of *Cyanobacteria*, *Tenericutes,* and *Saccharibacteria* was observed in healthy controls, whereas a higher abundance of *Elusimicrobia* was observed in bacterial keratitis cases. Furthermore, the study examined the association between fungal microbiome and bacterial keratitis. A significant increase in abundance of species of the genera *Aspergillus* and *Malassezia* in bacterial keratitis patients was observed, indicating that they could potentially be involved in increasing host susceptibility to bacterial infections of the cornea.

## 3. Specific Intestinal Commensals Linked to Ocular Immunity

*Bacteroides acidifaciens* is a gut commensal and a strict anaerobe linked to colonic immunoglobin A (IgA) production [25]. The crucial role of this bacteria in the ‘gut–eye axis’ of immune regulation was first discovered in an experiment investigating specific-pathogen-free Swiss Webster (SPF SW) mice treated with antibiotics cocktails and germ-free SW mice that underwent microbiota reconstitution [26]. Analysis of gut microbial communities revealed that *Bacteroides acidifaciens* was the most prominent species in SPF SW mice. Furthermore, *Bacteroides acidifaciens* was found to play a crucial role in promoting production of IgA transcripts in eye-associated lymphoid tissues and of secretory IgA in the colon, through induction of IL-1β. These processes are ultimately important in the generation of B-cell memory.

Changes in oral microbiome may potentially be associated with ocular morbidity, impacting ophthalmic conditions such as glaucoma and Sjogren syndrome (SS). Astafurov et al. conducted a clinical study exploring the association between the oral microbiome and glaucoma pathophysiology [27]. Results from the pyrosequencing of bacterial species indicated that both Gram-positive and Gram-negative bacterial loads were significantly higher in patients with glaucoma than in controls, meaning that peripheral (non-eye related) bacterial activity and/or products potentially played a role in the progression of glaucoma. An animal model was established to determine plausible mechanisms for the observation in the human study. It was found that upregulation toll-like receptor-4 (TLR-4) and the complement system was associated with a greater extent of neurodegeneration. Microglial activation in the affected tissues, associated with the altered oral bacterial loads, may also have contributed to the glaucoma pathophysiology. The results suggest that peripheral (non-eye related) bacterial activity and/or products may potentially have a role in glaucomatous optic neuropathy progression, as indicated by the finding of more prominent neurodegeneration and microglia activation of the retina and optic nerve with excessive bacterial loads.

Szymula et al. investigated whether peptides originating from oral and gut bacteria activated Sjögren’s syndrome Antigen A (SSA)/Ro60-reactive T cells [28]. Ro60/SSA is one of the major autoantigens targeted by autoimmunity in patients with SS and systemic lupus erythematosus (SLE) [29]. In this study, T cell hybridomas were generated from a mouse strain that lacked murine major histocompatibility complex (MHC) class II and expressed human leukocyte antigen DR3 (DRB1*0301 and DRA1*0101) transgenes [30]. Results showed that hyb-9.5 was activated by peptides originating from oral and gut microbes; hyb-26.4 by a peptide from the gut pathogen *Listeria grayi*, and hyb-34.14 by a peptide from the skin commensal *Acinetobacter johnsonii*. This provided a snapshot of cross-reactive microbial peptides capable of activating Ro60-reactive T cells, giving valuable clues to understanding the possible influence of oral and gut microbiota on autoimmunity.

Past reports implied that the gut bacterium *Prevotella copri* was involved in the pathogenesis of rheumatoid arthritis. A recent study indicated that it might also be involved in the initiation and/or perpetuation of anti-Ro60/SSA immune responses [31]. However, further experimental evidence is required to determine whether it is clearly a dysregulated immune response to normal microbiota that is involved in the pathogenesis of SS and SLE, rather than a specific infection.

Additionally, a peptide from the von Willebrand factor (VWF) type A domain protein, originating from the oral microbe *Capnocytophaga ochracea,* was found to be a potent activator of SSA/s [28]. VWF is a large multimeric glycoprotein present in plasma which allows platelets to coalesce and form a plug at injury sites [32]. Thus, the microbe may serve as a potential target in the oral mucosa as a potential trigger for initiating autoimmunity in SS or SLE.

## 4. Potential Ways for Therapeutic Manipulation of Intestinal Microbiota

### 4.1. Antibiotics Treatment

Minocycline is a pleiotropic broad-spectrum tetracycline antibiotic, with anti-inflammatory, anti-apoptotic, and immunomodulatory effects. If ingested orally, it has additional effects on gut microbiota [33]. It also has potent neuroprotective effects and has been used in the treatment of various neurodegenerative conditions [34]. Most importantly, minocycline intake has an excellent safety profile as demonstrated from a number of clinical trials. In experiments, minocycline treatment was shown to restore the relative abundance of *Ruminococcus bromii*, *Streptococcus hyointestinalis*, and *Desulfovibrio* sp. *ABHU2SB* and thus can remodel the gut microenvironment of EAU rats [35]. Oral administration of minocycline also conferred a higher abundance of intestinal *Parabacteroides goldsteinii* than placebo-treated EAU [36]. Furthermore, minocycline treatment significantly attenuated EAU disease severity [35]. However, this remains the only evidence of beneficial effects for antibiotic treatment in intestinal dysbiosis and ocular disease.

### 4.2. Probiotics Treatment

Kim et al. investigated the modulating effects of IRT-5 probiotics, a cocktail of five probiotic strains, on autoimmunity or alloimmunity in the eye, particularly in the context of autoimmunity of uveitis and dry eye and the alloimmunity of corneal transplantation [37]. IRT-5 probiotics consist of *Lactobacillus casei*, *Lactobacillus acidophilus*, *Lactobacillus reuteri*, *Bifidobacterium bifidum*, and *Streptococcus thermophilus*. Probiotics are living microorganisms that confer beneficial effects on the host organism by competing with potential pathogens or gut commensals [38]. Probiotics can be used as biotherapy to induce tolerance or modification of the immune system [38,39]. IRT-5 was shown to prevent the development of EAU and attenuate clinical manifestations of autoimmune dry eye models. Yet in alloimmunity, as found in corneal transplantation, IRT-5 treatment did not result in a prolonged survival of corneal allografts in animal models. In another experiment, IRT-5 treatment modified gut microbiome composition, downregulated antigen-presenting processes by immune cells, and improved clinical manifestations in a mouse model of autoimmune dry eye [40]. Quantitative proteome analysis showed downregulation of inflammatory cytokines, and upregulation of proteins associated with actin cytoskeleton organization, cell adhesion, and proteolysis. Network analysis, using differentially expressed proteins from the lacrimal glands, suggested that the ingestion of IRT-5 probiotics altered immunomodulatory and ionic transport-related protein expression in extra-orbital lacrimal glands [40]. Alternations in immunity was also noted in other parts of the body of the host. After IRT-5 treatment, CD11c^+^ cells in the spleen had extensive proteomic changes, where proteins involved in the antigen presentation pathway were significantly decreased. This suggests the impact of probiotic treatment on a number of immune pathways in the body [40]. Notably, this study was the first to identify proteomic changes in the lacrimal gland following alteration of the gut microbiome. Although the mechanism for this remains unclear [41], these observations provided a better understanding and future directions for investigating the gut–eye axis in dry eye disease.

## 5. Implications on Existing Therapy

Probiotics and antibiotics are currently available and viable therapeutic options for manipulation of the gut microbiome.

Treatment with IRT-5, a probiotic cocktail, has shown positive effects in preventing EAU development and ameliorating clinical manifestations in autoimmune dry eye in in vivo models. Given the effects of probiotics on host immunity, this provides further evidence to support probiotic treatment as a promising therapeutic strategy against inflammation-associated ocular disorders [37].

Regarding antibiotic treatment as a form of gut microbiome manipulation, there is evidence that such treatment may disrupt gut homeostasis and host immunity [42]. The combined administration of an antibiotic cocktail containing ampicillin, gentamicin, metronidazole, neomycin, and vancomycin reduces microbiome diversity and shifts the composition of gut microbiota [43,44], which in turn results in dysregulation of the host immune homeostasis and leads to an increased susceptibility to disease. On the other hand, however, the antibiotic minocycline is able to restore the architecture of gut microbiota in EAU rats, thereby ameliorating disease [35]. Thus, the jury is still out on the use of antibiotics for such purposes.

## 6. Current Techniques for Characterization of Gut Microbiome

Microbiological culture techniques have conventionally been used for identification and characterization of the ocular surface microbiome. However, these methods obtain a significantly less diverse profile compared with modern molecular techniques [45]. Moreover, in such techniques, species detection is significantly biased towards fast-growing microorganisms as they are more easily cultivated on a standard medium [46]. Nowadays, molecular techniques have circumvented problems arising from the use of standard culture methods and have helped enhance our knowledge on the microbial diversity of the gut.

Genomics-based detection and identification of microbial species, initially conducted in 2011 using 16S ribosomal ribonucleic acid (rRNA) polymerase chain reaction (PCR), are capable of identifying a more diverse bacterial population from a specific habitat when compared to culture-based methods [47]. Yet, one of the limitations of the 16S sequencing approach is its inability to specifically characterize commensals down to the level of bacterial species [48]. Hence, alternative approaches including transcriptome-based analysis or culturing methods are still essential for characterizing commensals.

More recently, shotgun metagenomics allow for simultaneous study of archaea, viruses, virophages, and eukaryotes [49,50], offering a greater potential for identification of strains, a more specific taxonomic and functional classification of sequences, and the discovery of new bacterial genes and genomic sequences. Although comparatively having limited taxonomical and functional resolution, 16S remains highly relevant in the analysis of large volumes of samples, i.e., large patient cohorts, and in longitudinal studies. This is because shotgun metagenomics is costly, owing to its increased resolution. Therefore, the decision between using the shotgun or the 16S approach for microbiome analyses should be dictated by the needs of the studies being conducted [51].

The advancement of technologies has led to the introduction of next-generation sequencing (NGS) platforms which allow for simultaneous sequencing of multiple targeted genomic regions in multiple samples [52]. In contrast to conventional sequencing methods, NGS analysis benefits from a reduced turnaround time of analysis and requires only small amounts of microbial DNA/RNA [52]. Despite the great potential of NGS platforms, bacterial contamination is routinely found in existing human-derived RNA-seq datasets during experiments. This likely arises from environmental sources. However, contamination can be isolated using a bioinformatics approach or statistical techniques. Meanwhile, the higher sensitivity of NGS platforms also necessitates more stringent sequencing and analysis protocols when handling clinical samples [53].

The majority of articles discussed in this review used genomic-based sequencing of bacterial 16S rRNA genes. There were a few studies that combined techniques for complementary purposes. In one study, the authors combined conventional culture technique with 16S rRNA sequencing [54], while in two other studies, shotgun sequencing was used to characterize individual microbes. In addition, one study used alkyne-functionalized D-alanine (alkDala) labeling to supplement their findings. This helps to detects metabolically active microbes rather than merely their nucleic acids. This technique is achieved by the labeling azide and alkyne-functionalized D-alanine that is present on various bacteria cell walls, which in turn can be visualized by click chemistry probes [55]. However, the drawback of alkDala labeling is that it is only able to detect the presence of live bacteria [56], which potentially leads to an incomplete presentation of the full microbial profile present in the tissue of interest.

## 7. Potential Challenges Ahead

Looking ahead, the study of the intestinal microbiome poses certain challenges. Sample collection and subsequent downstream processing guarantee the integrity and stability of the collected samples and ensure precision in DNA sequencing [57]. For clinical studies, environmental factors, such as dietary habits, drug consumption, intestinal motility, and stool frequency and consistency, are factors that should be considered in the study of gut microbiota composition [58]. In animal-based gut microbial studies, it is also essential to consider housing and dietary conditions in experiment designs [10]. Germ-free animals have extensive immunological defects, which may affect studies of ocular inflammatory disease. Interventions with antibiotics are also fraught with problems, as mentioned above. Cross-sectional human studies may be confounded by various factors such as environmental factors. Correlations or associations are also not proof of causation. Thus, randomized controlled clinical trials in ocular diseases are necessary for interventions such as fecal transplantations.

## 8. Future Directions

With established knowledge of the link between gut microbiota and immune-associated disease [59], it is important for future studies to further elucidate the function of gut commensals in shaping and maintaining host immune homeostasis. These studies also prompt the investigation of the impact of intestinal dysbiosis on neurodegeneration, in Alzheimer’s disease [60], and in Parkinson’s disease [61]. To overcome the challenges discussed, future experiments should focus on introducing only a small number of specific microbes, rather than induce global changes in the intestinal microbiome.

## 9. Conclusions

There is mounting evidence to support the important role of the gut microbiome in the pathogenesis of ophthalmic diseases, suggesting it is a promising target for both preventive measures and therapeutic treatments. An increasing number of studies have demonstrated that disruptions in gut microbiota impacted host immunity, as evidenced by altered T and B cell responses and cytokine secretion, as well as host metabolic responses. These changes in the immune system and metabolism in turn promoted the development of specific ophthalmic conditions in experimental settings. Further studies are however needed to better elucidate the underlying mechanisms involved.

## Figures and Tables

**Table 1:** The association between alterations in intestinal microbiome and the immunopathogenesis of specific ocular disorders.

Alterations in Intestinal Microbiota	Modifications in Host Immune Response and Metabolism	Specific Ocular Syndromes
Depletion of Roseburia and Subdoligranulum [5]	Reduction in regulatory T-cell responses and activation of immune-pathological T-effector responses [5]	Behçet’s disease (Promotion)
Germ-free environment [6]	Reduction in activation of T helper-17 cells in the intestine leading to amelioration of uveitis [6]	Autoimmune uveitis (Amelioration)
Reduction in bacterial phyla Firmicutes and Bacteroidetes, and the bacterial class, Alphaproteobacteria [7]	Increase in regulatory T-cells in lymphoid tissue and the retina and reduction in interleukin-17, interleukin-2 production, and effector T lymphocytes [7]	Autoimmune uveitis (Amelioration)
Increase in abundance of Anaerotruncus, Oscillibacter and Ruminococcus and Eubacterium ventriosum and depletion of Bacteroides eggerthii [8]	Reduction in fatty acid elongation and increased L-alanine fermentation, glutamine degradation, and arginine biosynthesis [8]	Age-related Macular degeneration (Promotion)
Increase in abundance of Negativicutes and reduction of the genus Oscillibacter and the phylum Bacteroidetes in AMD patients [9]	Positive correlation between higher abundance levels of Negativicutes and Complement factor H in age-related macula degeneration patients [9] Negative correlation between higher abundance levels of Bacteroides and Complement factor H in controls [9]	Age-related Macular degeneration (Promotion)
Reduction in proportion of Bacteroidetes [10]	Increased intestinal permeability and elevated circulating levels of interleukin-6, interleukin-1b, tumor necrosis factor-α, vascular endothelial growth factor-A, and the expression of inflammation-associated messenger ribonucleic acids [10]	Choroidal neovascularization (Promotion)
Increase in abundance of Dialister, Megasphaera, Faecalibacterium, Lachnospira, Ruminococcus and Mitsuokella and members of Firmicutes, Veillonellaceae, and Lachnospiraceae [11]	Reduction in levels of pathways linked to signaling molecules and G protein- coupled receptors [11]	Bacterial keratitis (Promotion)
Germ-free environment [12]	Increase in production of interleukin-12 by antigen-presenting cell in the lacrimal gland functional unit [12]	Sjögren-like lacrimal keratoconjunctivitis (Promotion)
Increase in abundance in Pseudobutyrivibrio, Escherichia/Shigella, Blautia, and Streptococcus, reduced abundance of Bacteroides, Parabacteroides, Faecalibacterium, and Prevotella [13]	Reduction in interleukin-13 and interleukin-13/Interferon-γ ratios and reduction in expression of the natural killer (NK)/natural killer T(NKT) associated integrin alpha 2 [13]	Sjögren-like lacrimal keratoconjunctivitis (Promotion)

## Data Availability

The authors agree to make all materials, data, and associated protocols promptly available to readers without undue qualifications in material transfer agreements.
